# Supplementary material for: Ten Years of Deformed Wing Virus (DWV) in Hawaiian Honey Bees (Apis mellifera), the Dominant DWV-A Variant Is Potentially Being Replaced by Variants with a DWV-B Coding Sequence
Source: Viruses. 2021 May 24;13(6):969. doi: 10.3390/v13060969 (PMC8225128; doi:10.3390/v13060969)
Supplement: Supplementary file 1 [file viruses-13-00969-s001.zip › viruses-1214302-supplementary.pdf]

Supplementary data

Includes the data of the samples used in the paper (Supplementary tables 1-3), the references used to construct figure 3 (Supplementary table 4) and a figure to illustrate the comparison of the treatment types (Supplementary figure 1)

Supplementary Table S1. Kauai samples, NEG/UD = Negative/Undetected, BL = Below the quantifiable threshold

								Average copy no.		Genome quivalent	
Sample ID	Collection date	Crush date	RNA conc.	Extraction date	Dilution factor	Type	Treatment	DWV-A RdRp	WV-B RdR	DWV-A RdRp	DWV-B RdRp
KCC44	04/12/2019	09/01/2020	624.0	24/11/2020	12.48	Managed	No	BL	2782.58	BL	2782.58
T7	15/11/2018	08/01/2020	233.7	24/11/2020	4.67	Feral	No	NEG/UD	BL	NEG/UD	BL
T4	29/08/2018	08/01/2020	283.3	24/11/2020	5.67	Feral	No	BL	649.58	BL	649.58
KRN4	04/12/2019	09/01/2020	693.1	24/11/2020	13.86	Managed	No	BL	NEG/UD	BL	NEG/UD
KRN5	04/12/2019	09/01/2020	502.4	24/11/2020	10.05	Managed	No	NEG/UD	NEG/UD	NEG/UD	NEG/UD
KNCW4	04/12/2019	10/01/2020	614.9	24/11/2020	12.30	Managed	No	264.16	NEG/UD	264.16	NEG/UD
KNLEW2	04/12/2019	10/01/2020	326.9	24/11/2020	6.54	Managed	No	NEG/UD	BL	NEG/UD	BL
KRN2	04/12/2019	10/01/2020	673.4	24/11/2020	13.47	Managed	No	317.90	BL	317.90	BL
KNCE5	04/12/2019	10/01/2020	916	24/11/2020	18.32	Managed	No	BL	BL	NEG/UD	BL
KNCW1 124-19	04/12/2019	10/01/2020	734.9	24/11/2020	14.70	Managed	No	NEG/UD	BL	NEG/UD	BL
KCC 73P	04/12/2019	10/01/2020	530.8	24/11/2020	10.62	Managed	No	NEG/UD	NEG/UD	NEG/UD	NEG/UD
KCC41	04/12/2019	13/01/2020	523.9	24/11/2020	10.48	Managed	No	BL	NEG/UD	BL	NEG/UD
KCC60	04/12/2019	13/01/2020	669.8	24/11/2020	13.40	Managed	No	NEG/UD	BL	NEG/UD	BL
KNCE4	04/12/2019	13/01/2020	653.3	24/11/2020	13.07	Managed	No	NEG/UD	BL	NEG/UD	BL
KNCE1	04/12/2019	13/01/2020	723.4	24/11/2020	14.47	Managed	No	NEG/UD	NEG/UD	NEG/UD	NEG/UD
KCC4	04/12/2019	13/01/2020	661	24/11/2020	13.22	Managed	No	NEG/UD	NEG/UD	NEG/UD	NEG/UD
KNCW3	04/12/2019	13/01/2020	1071	24/11/2020	21.42	Managed	No	NEG/UD	BL	NEG/UD	BL
KRN3	12/04/2019	13/01/2020	566.1	24/11/2020	11.32	Managed	No	NEG/UD	NEG/UD	NEG/UD	NEG/UD
KRN1	12/04/2019	13/01/2020	553.1	24/11/2020	11.06	Managed	No	BL	NEG/UD	BL	NEG/UD
KNCE3	12/04/2019	13/01/2020	419.4	24/11/2020	8.39	Managed	No	NEG/UD	BL	NEG/UD	BL
KNCE2	12/04/2019	13/01/2020	341.3	24/11/2020	6.83	Managed	No	NEG/UD	BL	NEG/UD	BL
KNCW5	12/04/2019	13/01/2020	685	24/11/2020	13.70	Managed	No	NEG/UD	BL	NEG/UD	BL

Supplementary Table S2. Oahu samples, NEG/UD = Negative/Undetected, BL = Below the quantifiable threshold

								Average copy no.		Genome equivalent	
Sample ID	Collection date	Crush date	RNA conc.	Extraction date	Dilution factor	Type	Treatment	DWV-A RdRp	DWV-B RdRp	DWV-A RdRp	DWV-B RdRp
SW-2	28/11/2019	21/01/2020	303.1	23/01/2020	6.06	Feral	No	3.93E+06	6.20E+06	4.77E+09	7.52E+09
TOL4	12/03/2019	09/01/2020	586.4	24/11/2020	11.73	Managed	Yes	2.05E+04	8.76E+04	4.81E+07	2.06E+08
TOL1	12/03/2019	09/01/2020	444.4	24/11/2020	8.89	Managed	Yes	2.79E+06	1.48E+05	4.97E+09	2.63E+08
TOL2	12/03/2019	10/01/2020	575.8	24/11/2020	11.52	Managed	Yes	6.07E+05	4.73E+05	1.40E+09	1.09E+09
TOL3	12/03/2019	10/01/2020	725.2	24/11/2020	14.50	Managed	Yes	7.35E+05	6.77E+05	2.13E+09	1.96E+09
TOL5	12/03/2019	13/01/2020	496.9	24/11/2020	9.94	Managed	Yes	1.78E+05	5.07E+05	3.53E+08	1.01E+09
TOLs	03/12/2019	08/01/2020	155.3	24/11/2020	3.11	Feral	No	4.24E+05	5.71E+06	2.63E+08	3.55E+09
UH42	29/11/2019	08/01/2020	460.3	24/11/2020	9.21	Managed	Yes	7.50E+04	2.48E+05	1.38E+08	4.56E+08
UH132	29/11/2019	08/01/2020	693.6	24/11/2020	13.87	Managed	Yes	3.34E+05	5.64E+05	9.28E+08	1.56E+09
UH127	29/11/2019	08/01/2020	754.7	24/11/2020	15.09	Managed	Yes	2.89E+06	2.36E+06	8.72E+09	7.12E+09
UH140	29/11/2019	09/01/2020	639.8	24/11/2020	12.80	Managed	Yes	1.09E+04	5.42E+04	2.80E+07	1.39E+08
UH107	29/11/2019	09/01/2020	700.3	24/11/2020	14.01	Managed	Yes	2.10E+06	1.38E+06	5.89E+09	3.87E+09
T14	16/06/2019	09/01/2020	433.7	24/11/2020	8.67	Feral	No	8.72E+05	2.45E+06	1.51E+09	4.25E+09
OPATCR	01/11/2019	21/01/2020	646.1	23/01/2020	12.92	Managed	No	4.22E+06	6.14E+06	1.09E+10	1.59E+10
OPATC	01/11/2019	21/01/2020	456.8	23/01/2020	9.14	Managed	No	4.34E+05	2.53E+03	7.93E+08	4.62E+06
OPATC SCOT	01/11/2019	21/01/2020	352.8	23/01/2020	7.06	Managed	No	1.23E+06	5.05E+05	1.73E+09	7.13E+08
PATC	20/11/2019	16/01/2020	396.3	23/01/2020	7.93	Managed	No	6.47E+05	1.82E+04	1.03E+09	2.89E+07
OPATCM	20/11/2019	22/01/2020	581.6	23/01/2020	11.63	Managed	No	1.73E+05	1.07E+05	4.02E+08	2.50E+08
OUGCP20	01/11/2019	21/01/2020	515.9	23/01/2020	10.32	Managed	Yes	5.38E+06	2.67E+05	1.11E+10	5.50E+08
UGUPC5	20/11/2019	16/01/2020	443.6	23/01/2020	8.87	Managed	Yes	1.76E+07	1.41E+06	3.12E+10	2.50E+09
OUGCPC3	21/11/2019	21/01/2020	746.2	23/01/2020	14.92	Managed	Yes	3.44E+06	2.46E+06	1.03E+10	7.36E+09
OUGCWhite	20/11/2019	22/01/2020	511.5	23/01/2020	10.23	Managed	Yes	1.29E+06	1.51E+06	2.63E+09	3.10E+09
OUGCJ	20/11/2019	16/01/2020	630.5	23/01/2020	12.61	Managed	Yes	8.35E+04	7.24E+04	2.11E+08	1.83E+08
ODAL1	01/11/2019	21/01/2020	747.4	23/01/2020	14.95	Managed	No	4.73E+04	6.79E+03	1.41E+08	2.03E+07
ODAL4	21/11/2019	21/01/2020	689.8	23/01/2020	13.80	Managed	No	8.51E+03	2.03E+04	2.35E+07	5.59E+07
ODAL2	21/11/2019	22/01/2020	549.8	23/01/2020	11.00	Managed	No	3.59E+06	3.21E+04	7.90E+09	7.07E+07
ODAL5	21/11/2019	22/01/2020	629.0	23/01/2020	12.58	Managed	No	1.42E+06	4.19E+05	3.58E+09	1.06E+09
ODAL3	21/11/2019	22/01/2020	584.1	23/01/2020	11.68	Managed	No	7.82E+05	1.88E+06	1.83E+09	4.40E+09
ODEN6AA4	21/11/2019	21/01/2020	715.3	23/01/2020	14.31	Managed	No	1.90E+06	3.46E+05	5.45E+09	9.89E+08
ODEN5E3	21/11/2019	21/01/2020	713.1	23/01/2020	14.26	Managed	No	9.06E+05	1.35E+06	2.58E+09	3.84E+09
ODEN8C2	21/11/2019	22/01/2020	583.4	23/01/2020	11.67	Managed	No	1.65E+06	2.74E+06	3.85E+09	6.39E+09
ODEN7AI	21/11/2019	22/01/2020	750.8	23/01/2020	15.02	Managed	No	1.82E+06	1.40E+06	5.46E+09	4.19E+09
ODEN6A2	21/11/2019	22/01/2020	803.9	23/01/2020	16.08	Managed	No	1.34E+05	2.21E+05	4.30E+08	7.10E+08
4CI 3	29/11/2019	22/01/2020	729.3	23/01/2020	14.59	Managed	No	1.20E+07	9.07E+04	3.50E+10	2.65E+08
4CI 4	29/11/2019	22/01/2020	820.9	23/01/2020	16.42	Managed	No	562.22	4849.26	1.85E+06	1.59E+07
4CI 5	29/11/2019	22/01/2020	499.7	23/01/2020	9.99	Managed	No	512.31	3420.49	1.02E+06	6.84E+06
4CI 1	29/11/2019	22/01/2020	450.9	23/01/2020	9.02	Managed	No	BL	625.15	BL	1.13E+06
4CI 2	29/11/2019	22/01/2020	725	23/01/2020	14.50	Managed	No	NEG/UD	3191.33	NEG/UD	9.25E+06
IOD4 1	29/11/2019	22/01/2020	745.0	23/01/2020	14.90	Managed	No	2.30E+06	4.22E+06	6.84E+09	1.26E+10
IOD4 2	29/11/2019	22/01/2020	786.3	23/01/2020	15.73	Managed	No	261.25	2630.45	8.22E+05	8.27E+06
IOD4 3	29/11/2019	22/01/2020	824.6	23/01/2020	16.49	Managed	No	BL	3385.47	BL	1.12E+07
IOD4 4	29/11/2019	22/01/2020	411.8	23/01/2020	8.24	Managed	No	259.37	2887.09	4.27E+05	4.76E+06
IOD4 5	29/11/2019	22/01/2020	833.2	23/01/2020	16.66	Managed	No	BL	3277.73	BL	1.09E+07
T13	08/10/2018	08/01/2020	295.4	24/11/2020	5.908	Feral	No	1.58E+05	7.40E+04	1.87E+08	8.74E+07
T2	15/06/2018	08/01/2020	355.5	24/11/2020	7.11	Feral	No	7.84E+04	3.16E+04	1.12E+08	4.50E+07
T1	11/01/2018	08/01/2020	438.1	24/11/2020	8.762	Feral	No	5.21E+03	1.14E+05	9.13E+06	2.00E+08
T3	13/07/2018	08/01/2020	366.8	24/11/2020	7.336	Feral	No	3.20E+03	2.26E+03	4.70E+06	3.31E+06
T12	18/05/2018	08/01/2020	393.3	24/11/2020	7.866	Feral	No	9.11E+04	2.92E+05	1.43E+08	4.60E+08
T12	15/06/2018	08/01/2020	472.2	24/11/2020	9.444	Feral	No	3.39E+05	9.00E+04	6.41E+08	1.70E+08
T4	09/07/2018	08/01/2020	536.9	24/11/2020	10.738	Feral	No	2.56E+05	2.29E+05	5.50E+08	4.92E+08
T4	10/08/2018	08/01/2020	356.6	24/11/2020	7.132	Feral	No	6.08E+05	1.70E+05	8.67E+08	2.42E+08

Supplementary Table S3. Big Island samples, NEG/UD = Negative/Undetected, BL = Below the

								Average copy no.		Genome quivalent	
Sample ID	Collection date	Crush date	RNA conc.	Extraction date	Dilution factor	Type	Treatment	DWV-A RdRp	DWV-B RdRp	DWV-A RdRp	DWV-B RdRp
SP1	25/11/2019	15/01/2020	699.7	23/01/2020	13.99	Managed	Yes	1.78E+06	2.38E+04	4.97E+09	6.65E+07
SP2	25/11/2019	15/01/2020	745.1	24/11/2020	14.90	Managed	Yes	2.10E+06	3.08E+07	6.26E+09	9.19E+10
SP3	25/11/2019	09/01/2020	565.3	24/11/2020	11.31	Managed	Yes	4.67E+04	3.11E+06	1.06E+08	7.03E+09
SP4	29/11/2019	09/01/2020	709.2	24/11/2020	14.18	Managed	Yes	3.76E+06	9.52E+07	1.07E+10	2.70E+11
SP5	25/11/2019	14/01/2020	592.9	24/11/2020	11.86	Managed	Yes	8.10E+05	4.51E+07	1.92E+09	1.07E+11
SB4	24/11/2019	15/01/2020	844.8	23/01/2020	16.90	Managed	Yes	9.64E+06	6.66E+06	3.26E+10	2.25E+10
SB3	24/11/2019	15/01/2020	672.6	23/01/2020	13.45	Managed	Yes	1.06E+04	8.58E+03	2.85E+07	2.31E+07
SB2	24/11/2019	16/01/2020	910.8	23/01/2020	18.22	Managed	Yes	3.23E+06	1.49E+06	1.18E+10	5.44E+09
SB5	24/11/2019	16/01/2020	882.0	23/01/2020	17.64	Managed	Yes	4.24E+04	2.47E+03	1.50E+08	8.72E+06
SB1	24/11/2019	09/01/2020	796.2	24/11/2020	15.92	Managed	Yes	4.74E+06	2.54E+07	1.51E+10	8.10E+10
GAR1	23/11/2019	22/01/2020	228.0	23/01/2020	4.56	Managed	Yes	4.87E+04	1.55E+06	4.44E+07	1.42E+09
GAR4	23/11/2019	22/01/2020	457.3	23/01/2020	9.15	Managed	Yes	5.57E+05	4.96E+05	1.02E+09	9.06E+08
GAR2	23/11/2019	14/01/2020	543.5	24/11/2020	10.87	Managed	Yes	6.87E+06	3.19E+07	1.49E+10	6.93E+10
GAR3	23/11/2019	16/01/2020	509.2	23/01/2020	10.18	Managed	Yes	6.80E+06	1.77E+06	1.38E+10	3.61E+09
GAR5	23/11/2019	14/01/2020	318.9	24/11/2020	6.38	Managed	Yes	9.29E+06	7.23E+07	1.19E+10	9.22E+10
KR3	23/11/2019	21/01/2020	719.8	23/01/2020	14.40	Managed	Yes	3.32E+03	6.60E+04	9.57E+06	1.90E+08
KR5	23/11/2019	14/01/2020	503.7	24/11/2020	10.07	Managed	Yes	2.82E+05	6.47E+06	5.69E+08	1.30E+10
KR7	23/11/2019	16/01/2020	480.5	23/01/2020	9.61	Managed	Yes	1.05E+06	5.37E+06	2.01E+09	1.03E+10
KR4	23/11/2019	14/01/2020	526.2	24/11/2020	10.52	Managed	Yes	BL	BL	BL	BL
KR2	23/11/2019	14/01/2020	675.0	24/11/2020	13.50	Managed	Yes	473.29	71110.55	1.28E+06	1.92E+08
KR6	23/11/2019	15/01/2020	550.5	23/01/2020	11.01	Managed	Yes	1.63E+06	8.62E+03	3.59E+09	1.90E+07
KR1	23/11/2019	14/01/2020	437.4	24/11/2020	8.75	Managed	Yes	1.65E+04	4.59E+04	2.88E+07	8.03E+07
RON1	26/11/2019	15/01/2020	985.9	23/01/2020	19.72	Managed	Yes	4.77E+07	9.42E+06	1.88E+11	3.72E+10
RON5	26/11/2019	14/01/2020	394.7	24/11/2020	7.89	Managed	Yes	1445.87	BL	2.28E+06	BL
RON2	26/11/2019	14/01/2020	649.7	24/11/2020	12.99	Managed	Yes	1.07E+07	5.51E+03	2.78E+10	1.43E+07
RON4	26/11/2019	14/01/2020	864.2	24/11/2020	17.28	Managed	Yes	5.63E+04	2.87E+03	1.94E+08	9.91E+06
RON3	26/11/2019	16/01/2020	657.9	23/01/2020	13.16	Managed	Yes	4.41E+05	9.53E+03	1.16E+09	2.51E+07
DA1	24/11/2019	15/01/2020	659.0	23/01/2020	13.18	Managed	Yes	2.83E+07	9.33E+06	7.45E+10	2.46E+10
DA2	24/11/2019	15/01/2020	700.6	24/11/2020	14.01	Managed	Yes	BL	352279.44	BL	9.87E+08
DA3	24/11/2019	21/01/2020	640.1	23/01/2020	12.80	Managed	Yes	7.20E+05	6.51E+03	1.84E+09	1.67E+07
DA5	24/11/2019	16/01/2020	553.4	23/01/2020	11.07	Managed	Yes	1.55E+03	6.09E+06	3.43E+06	1.35E+10
DA4	24/11/2019	15/01/2020	385.0	23/01/2020	7.70	Managed	Yes	2.39E+07	1.67E+05	3.67E+10	2.57E+08
DT1	25/11/2019	15/01/2020	797.8	23/01/2020	15.96	Managed	Yes	3.14E+03	6.81E+06	1.00E+07	2.17E+10
DT2	25/11/2019	14/01/2020	684.8	24/11/2020	13.70	Managed	Yes	7.83E+06	2.64E+06	2.14E+10	7.24E+09
DT3	25/11/2019	15/01/2020	705.7	23/01/2020	14.11	Managed	Yes	4.27E+05	8.41E+04	1.20E+09	2.37E+08
DT4	25/11/2019	15/01/2020	970.5	24/11/2020	19.41	Managed	Yes	931.92	6043.39	2.22E+06	9.99E+05
DT5	25/11/2019	09/01/2020	860.6	24/11/2020	17.21	Managed	Yes	645.84	BL	3.62E+06	BL
VAN LW1	26/11/2019	15/01/2020	663.1	23/01/2020	13.26	Managed	Yes	3.57E+06	9.05E+06	9.47E+09	2.40E+10
VAN17	26/11/2019	15/01/2020	619.4	24/11/2020	12.39	Managed	Yes	283.38	1170.97	7.02E+05	2.90E+06
VAN8	26/11/2019	14/01/2020	777	24/11/2020	15.54	Managed	Yes	174.69	BL	5.43E+05	BL
VAN L25	26/11/2019	15/01/2020	810	24/11/2020	16.20	Managed	Yes	121.06	3263.12	3.92E+05	1.06E+07
VAN4	26/11/2019	15/01/2020	1067.3	24/11/2020	21.35	Managed	Yes	3.77E+05	2.81E+03	1.61E+09	1.20E+07
SW1	24/11/2019	16/01/2020	578.4	23/01/2020	11.57	Feral	No	2.49E+07	1.10E+07	5.77E+10	2.54E+10

quantifiable threshold

Table S4. DWV world map references

Country/Region	Source reference
Argentina – Buenos Aires and Santa Fe province	(Brasesco et al., 2020)
Australia	(Roberts, Anderson, & Durr, 2017)
Brazil	(de Souza, Kevill, Correia-Oliveira, de Carvalho, & Martin, 2019)
Chile	(Riveros et al., 2019)
China	(Diao et al., 2019)
Cuba	(Luis et al., 2020)
Ethiopia – Tigray	(Gebremedhn et al., 2020)
Fernando de Noronha	(Brettell & Martin, 2017)
France	(Manley et al., 2019)
Germany	(Natsopoulou et al., 2017)
Hawaii	This study, (Brettell, Schroeder, & Martin, 2020)
Kenya	(Ongus, Fombong, Irungu, Masiga, & Raina, 2018)
Other	(Beaurepaire et al., 2020)
Other	(Wilfert et al., 2016)
Papua new guinea	(Roberts, Simbiken, Dale, Armstrong, & Anderson, 2020)
South Africa	(de Souza, Allsopp, & Martin, 2021)
Tunisia	(Abdi et al., 2018)
Turkey	(Tozkar, Kence, Kence, Huang, & Evans, 2015)
UK	(Kevill et al., 2019)
Uruguay	(Mendoza et al., 2020)
USA	(Kevill et al., 2019)

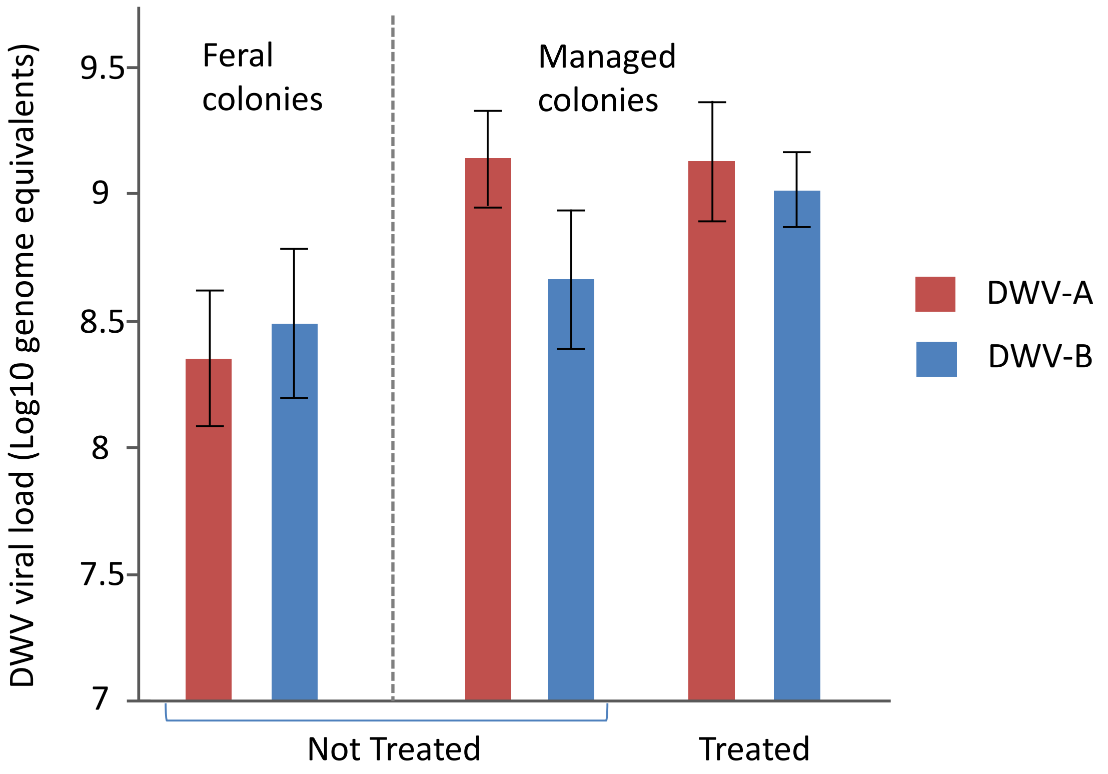

Supplementary Figure S1. Average DWV-A and –B loads in colonies of different treatment type from Oahu with bars showing the standard error.

References

Abdi, K., Belguith, K., Hamdi, C., Souissi, Y., Essanaa, J., Dridi, W., . . . Cherif, A. (2018). Parasites-Iflavirus association and emergence of three master variants of DWV affecting *Apis mellifera intermissa* in Tunisian apiaries. *Bulletin of Insectology, 71*(2), 273-282.

Beaurepaire, A., Piot, N., Doublet, V., Antunez, K., Campbell, E., Chantawannakul, P., . . .Dalmon, A. (2020). Diversity and global distribution of viruses of the western honey bee, *Apis mellifera*. *Insects*, *11*(4), 239. doi: 10.3390/insects11040239

Brasesco, C., Quintana, S., Di Geronimo, V., Genchi Garcia, M. L., Sguazza, G., Bravi, M. E., . . . Maggi, M. (2020). Deformed wing virus type a and b in managed honeybee colonies of Argentina. *Bull Entomol Res*, 1-11. doi:10.1017/S000748532000036X

Brettell, L. E., & Martin, S. J. (2017). Oldest Varroa tolerant honey bee population provides insight into the origins of the global decline of honey bees. *Sci Rep, 7*, 45953. doi:10.1038/srep45953

Brettell, L. E., Schroeder, D. C., & Martin, S. J. (2020). RNAseq of Deformed Wing Virus and Other Honey Bee-Associated Viruses in Eight Insect Taxa with or without *Varroa* Infestation. *Viruses, 12*(11). doi:10.3390/v12111229

de Souza, F. S., Allsopp, M., & Martin, S. J. (2021). Deformed wing virus prevalence and load in honeybees in South Africa. *Arch. Virol.*, *166*, 237-241.

de Souza, F. S., Kevill, J. L., Correia-Oliveira, M. E., de Carvalho, C. A. L., & Martin, S. J. (2019). Occurrence of Deformed wing virus variants in the stingless bee *Melipona subnitida* and

honey bee *Apis mellifera* populations in Brazil. *J. Gen. Virol., 100*(2), 289-294. doi:10.1099/jgv.0.001206

Diao, Q., Yang, D., Zhao, H., Deng, S., Wang, X., Hou, C., & Wilfert, L. (2019). Prevalence and population genetics of the emerging honey bee pathogen DWV in Chinese apiculture. *Sci Rep, 9*(1), 12042. doi:10.1038/s41598-019-48618-y

Gebremedhn, H., Deboutte, W., Schoonvaere, K., Demaeght, P., De Smet, L., Amssalu, B., . . . de Graaf, D. C. (2020). Metagenomic Approach with the NetoVIR Enrichment Protocol Reveals Virus Diversity within Ethiopian Honey Bees (*Apis mellifera simensis*). *Viruses, 12*(11). doi:10.3390/v12111218

Kevill, J. L., de Souza, F. S., Sharples, C., Oliver, R., Schroeder, D. C., & Martin, S. J. (2019). DWV-A Lethal to Honey Bees (*Apis mellifera*): A Colony Level Survey of DWV Variants (A, B, and C) in England, Wales, and 32 States across the US. *Viruses, 11*(5). doi:10.3390/v11050426

Luis, A. R., García, C. A. Y., Invernizzi, C., Branchiccela, B., Piñeiro, A. M. P., Morfi, A. P., . . . Antúnez, K. (2020). Nosema ceranae and RNA viruses in honey bee populations of Cuba. *Journal of Apicultural Research, 59*(4), 468-471. doi:10.1080/00218839.2020.1749451

Manley, R., Temperton, B., Doyle, T., Gates, D., Hedges, S., Boots, M., & Wilfert, L. (2019). Knock-on community impacts of a novel vector: spillover of emerging DWV-B from *Varroa*-infested honeybees to wild bumblebees. *Ecol Lett, 22*(8), 1306-1315. doi:10.1111/ele.13323

Mendoza, Y., Tomasco, I., Antunez, K., Castelli, L., Branchiccela, B., Santos, E., & Invernizzi, C. (2020). Unraveling honey bee–*Varroa destructor* interaction: Multiple factors involved in differential resistance between two uruguayan populations. *Vet. Sci., 7*(3).

Natsopoulou, M. E., McMahon, D. P., Doublet, V., Frey, E., Rosenkranz, P., & Paxton, R. J. (2017). The virulent, emerging genotype B of Deformed wing virus is closely linked to overwinter honeybee worker loss. *Sci Rep, 7*(1), 5242. doi:10.1038/s41598-017-05596-3

Ongus, J. R., Fombong, A. T., Irungu, J., Masiga, D., & Raina, S. (2018). Prevalence of common honey bee pathogens at selected apiaries in Kenya, 2013/2014. *International Journal of Tropical Insect Science, 38*(1), 58-70. doi:10.1017/S1742758417000212

Riveros, G., Arismendi, N., Zapata, N., Evans, D., Pérez, I., Aldea, P., & Vargas, M. (2019). Occurrence, prevalence and viral load of deformed wing virus variants in *Apis mellifera* colonies in Chile. *Journal of Apicultural Research, 59*(1), 63-68. doi:10.1080/00218839.2019.1670993

Roberts, J. M. K., Anderson, D. L., & Durr, P. A. (2017). Absence of deformed wing virus and *Varroa destructor* in Australia provides unique perspectives on honeybee viral landscapes and colony losses. *Sci. Rep., 7*(1), 6925. doi:10.1038/s41598-017-07290-w

Roberts, J. M. K., Simbiken, N., Dale, C., Armstrong, J., & Anderson, D. L. (2020). Tolerance of honey bees to *Varroa* mite in the absence of Deformed wing virus. *Viruses, 12*(5). doi:10.3390/v12050575

Tozkar, C. O., Kence, M., Kence, A., Huang, Q., & Evans, J. D. (2015). Metatranscriptomic analyses of honey bee colonies. *Front Genet, 6*, 100. doi:10.3389/fgene.2015.00100

Wilfert, L., Long, G., Leggett, H. C., Schmid-Hempel, P., Butlin, R., Martin, S. J. M., & Boots, M. (2016). Deformed wing virus is a recent global epidemic in honeybees driven by Varroa mites. *Science, 351*(6273), 594. doi:10.1126/science.aac9976
